# *Mycoplasma haemolamae* and intestinal parasite relationships with erythrocyte variables in clinically healthy alpacas and llamas

**DOI:** 10.1111/jvim.15596

**Published:** 2019-08-27

**Authors:** Lisa C. Viesselmann, Ricardo Videla, John Schaefer, Aly Chapman, Heidi Wyrosdick, Deanna M. W. Schaefer

**Affiliations:** ^1^ Department of Biomedical and Diagnostic Sciences University of Tennessee Knoxville Tennessee; ^2^ Department of Large Animal Clinical Sciences University of Tennessee Knoxville Tennessee

**Keywords:** anemia, camelid, endoparasite, fecal egg count, hemoparasite

## Abstract

**Background:**

*Mycoplasma haemolamae* (Mhl) and gastrointestinal nematodes can cause anemia in camelids. Control programs aim to suppress parasitism without promoting anthelminthic resistance, but few evidence‐based guidelines define acceptable parasite loads in camelids.

**Hypothesis/Objectives:**

In clinically healthy nonanemic camelids, compare erythrocyte variables to Mhl real‐time PCR status and to fecal egg count (FEC). Determine the FEC threshold above which erythrocyte variables are consistently below reference interval medians.

**Animals:**

One hundred fourteen client‐owned adult alpacas and llamas.

**Methods:**

In a cross‐sectional study, whole blood in ethylenediaminetetraacetic acid (EDTA) was assessed for packed cell volume (PCV) by centrifugation, erythrocyte count (RBC), and hemoglobin concentration (HGB) using an ADVIA120 analyzer, and Mhl using real‐time PCR. Trichostrongyle eggs per gram (epg) were counted by modified McMaster test on freshly collected feces. Significant differences in erythrocyte variables based on Mhl status and FEC thresholds were assessed by independent *t* test and one‐way ANOVA, respectively.

**Results:**

Packed cell volume, RBC, and HGB were not significantly different between Mhl‐positive and Mhl‐negative animals, but were significantly lower in animals with FEC >1000 epg compared to those with <500 epg. All animals with FEC >600 epg had RBC and HGB below the reference interval median. All animals with FEC >750 epg had PCV below the reference interval median.

**Conclusions and Clinical Importance:**

In healthy nonanemic camelids, positive Mhl PCR is not associated with lower erythrocyte variables and such animals may not warrant treatment. Fecal egg count >600‐750 epg has a negative effect on erythrocyte variables, and may be a guide for deworming protocols.

**Abbreviations**
BCS: body condition score
C_T_: cycle threshold
EDTA: ethylenediaminetetraacetic acid
epg: eggs per gram
FAMACHA: Faffa Malan Chart
FEC: fecal egg count
HGB: hemoglobin concentration
Mhl: *Mycoplasma haemolamae*
RBC: red blood cell/erythrocyte count
UTVMC: University of Tennessee Veterinary Medical Center

## 1 INTRODUCTION

*Mycoplasma haemolamae* (Mhl) is a hemotropic bacterium that can cause clinically apparent anemia in alpacas and llamas. Clinically relevant anemia is reported most often in infected animals that are immunosuppressed, stressed, debilitated, or suffering from a concurrent illness, but organisms still can be observed on peripheral blood smears from some clinically healthy animals.^1^ *Haemonchus contortus* is a trichostrongylid nematode that resides in the third gastric compartment (C3). It is the most common and clinically important endoparasite of alpacas and llamas, and is a common cause of clinically relevant anemia.^2^, ^3^

The gold standard test for diagnosis of Mhl is a real‐time PCR assay that is specific for detection of the organism's 16S rRNA gene.^4^, ^5^ However, bacteremia in infected animals is transient and cyclical, and it therefore can be difficult to identify Mhl as an etiologic agent in animals with anemia.^6^ Additionally, most infections are subclinical, resulting in a carrier state that persists despite antibiotic treatment of infected animals.^1^ Previous studies have reported that infection with Mhl (based on positive real‐time PCR results) is not significantly associated with low PCV.^5^, ^7^

The gold standard for diagnosis and quantification of intestinal nematodes is adult worm counts at necropsy. Antemortem, trichostrongylid infections can be diagnosed and semi‐quantified using the fecal egg count test (FEC), which measures the number of trichostrongyle eggs per gram (epg) of feces. However, this test cannot differentiate *H. contortus* eggs from other trichostrongyle eggs (eg, *Teladorsagia circumcincta, Trichostrongylus axei*).^3^ In addition, no established thresholds define clinically relevant FECs in camelids. The FEC has been shown to be negatively correlated with PCV and other erythrocyte variables (erythrocyte count [RBC], hemoglobin concentration [HGB]) in clinically ill alpacas and llamas.^3^, ^8^, ^9^ However, no documentation has been published of any correlation between FEC and erythrocyte variables in clinically healthy, nonanemic camelids. Tools used to identify animals in need of deworming treatment, such as the Faffa Malan Chart system (FAMACHA©),^10^ only identify animals that are already anemic, potentially missing nonanemic animals with high nematode burdens. Conversely, deworming all animals in a herd regardless of their nematode burden promotes parasite resistance to anthelminthic medications.

Because PCV is only 1 index of erythrocyte mass, a goal of our study was to perform a more comprehensive assessment of the relationship between parasitism and erythrocyte variables, including PCV, RBC, and HGB. We hypothesized that clinically healthy, nonanemic animals that were PCR‐positive for Mhl would not have significantly lower results for erythrocyte variables compared to those with negative PCR results, but that erythrocyte variables would be lower in animals with high FEC compared to those with low FEC. Additionally, we hypothesized that there would be an FEC threshold above which erythrocyte variables in clinically healthy, nonanemic alpacas and llamas would be consistently below the reference interval medians, suggesting that although the animals were not clinically anemic, they may still have adverse hematologic effects and would benefit from parasite control strategies.

## 2 MATERIALS AND METHODS

### 2.1 Patient population and sample collection

Venous whole blood was collected from clinically healthy adult (≥ 1‐year‐old) alpacas and llamas from 11 farms in eastern Tennessee between July and October 2015. Blood also was collected from 2 blood donor alpacas housed by the University of Tennessee during the same time period. Health was defined as a lack of clinically relevant abnormalities on physical examination as determined by an experienced veterinarian, a FAMACHA score ≤ 3/5,^10^ a body condition score (BCS) ≥ 2.5/5,^11^ and no history of illness or injury within the previous 3 months. All examinations and sample collections took place on the farms, and informed client consent was obtained for all procedures (approved Institutional Animal Care and Use protocol 2298‐0914).

Samples were collected from no more than 12 and no fewer than 7 animals per farm. Individual and herd histories were obtained, including species, age, sex, pregnancy and breeding status, housing, diet, vaccines, and parasite control practices. After examination of each animal, a maximum of 10 mL of blood was collected from an external jugular vein using a 12 mL plastic syringe (Covidien LLC, Mansfield, MA) attached to a 20 gauge, 1.5‐in. needle (Covidien LLC, Mansfield, MA). Blood was placed in a 4 mL K_2_EDTA tube (Becton Dickinson, Franklin Lakes, NJ) and transported to the clinical pathology laboratory at the University of Tennessee Veterinary Medical Center (UTVMC) in a cooler that was maintained at 4°C until arrival. Blood samples were analyzed promptly (all analyses were performed within 6 hours of sample collection) by trained laboratory personnel. Samples were evaluated for appropriate filling of the EDTA tube, and for clot formation, gross lipemia, or both. Underfilled, clotted, or lipemic samples were excluded. A CBC was performed on each sample using the ADVIA120 hematology instrument^12^ (Siemens Healthcare Diagnostics, Tarrytown, NY), and samples were excluded if the PCV, HGB, or RBC were below the UTVMC reference intervals for camelids.

Manual PCVs were determined after 1 minute of centrifugation in a rapid fixed angle head microhematocrit centrifuge (HemataStat II, EKF Diagnostics, Boerne, TX). The centrifuge's read function was used to determine PCV; this function is calibrated for the microhematocrit's centrifuge speed. Proper microhematocrit centrifuge function was verified using an electronic tachometer before beginning the study, and a series of test centrifugations of camelid blood (data not shown) confirmed that there was no clinically relevant difference in PCV with centrifugation times >1 minute. Determinations of PCV were made manually and using the ADVIA120 analyzer by a licensed medical technologist trained in instrument use and following laboratory standard operating procedures. The ADVIA120 underwent daily quality control using 3‐level quality control material (OPTIpoint, Siemens Healthcare Diagnostics, Tarrytown, NY).

A minimum of 1 blood smear was prepared from each sample, and reviewed by a medical technologist. Blood smear review included a leukocyte differential count, erythrocyte morphology review, and manual platelet estimate. Each blood smear also was reviewed for Mhl parasites by a board‐certified veterinary clinical pathologist.

A fresh fecal sample was collected digitally from the rectum of each animal by trained personnel, placed in an individual, clean plastic bag, and transported in a cooler to the UTVMC parasitology laboratory.

### 2.2 *M. haemolamae* and intestinal parasite testing

After performing the CBC, the remaining EDTA‐anticoagulated whole blood from each animal was stored at −20°C for up to 30 days. Extraction of DNA and real‐time PCR for Mhl were performed using previously described methods^5^ (Applied Biosystems StepOne, ThermoFisher Scientific, Waltham, MA). Positive and negative control samples were included in each run. Individual samples were classified as positive or negative based on their cycle threshold (C_T_), with samples having a C_T_ ≤ 35.0 classified as positive.

The FEC was performed on each fecal sample using the modified McMaster's test, as previously described for alpacas and llamas.^13^ Briefly, 2 g of feces were mixed with 28 mL of sodium nitrate flotation solution with a specific gravity of 1.200. The mixture was loaded onto a McMaster's slide, and examined under light microscopy after 5 minutes of flotation. The number of strongylid eggs in both chambers was counted at 100× total magnification, and the total number counted in each sample was multiplied by 50 to obtain results in epg of feces. An egg count of 0 was reported as <50 epg. Because egg morphology is insufficient to differentiate *Haemonchus* spp. from other trichostrongyle species, nematode larval cultures also were performed on a subset of the fecal samples to further evaluate for the presence of *Haemonchus contortus*.

### 2.3 Statistical analysis

Statistical analysis was performed using MedCalc Statistical Software version 17.4 (MedCalc Software bvba, Ostend, Belgium; http://www.medcalc.org; 2017). The distributions of erythrocyte variables (PCV, RBC, HGB) in *M. haemolamae*‐positive (Mhl‐positive) and *M. haemolamae*‐negative (Mhl‐negative) animals were assessed for normality using the D'Agostino‐Pearson test. The means and medians of the 2 groups were compared using an independent *t* test or Mann‐Whitney rank sum test. Significance was based on a *P* value <.05. The sample population also was empirically stratified into groups with low (<500 epg), moderate (500‐1000 epg), and high (>1000 epg) FEC. Erythrocyte variables were compared among the different FEC strata using a 1‐way ANOVA or Kruskal‐Wallis test, and significance was based on a *P* value <.05. Comparison of erythrocyte variables also was performed between Mhl‐positive and Mhl‐negative animals in the different FEC strata.

## 3 RESULTS

### 3.1 Animals

Anticoagulated whole blood and fecal samples were collected from 125 clinically healthy adult New World camelids. Seven animals were excluded because of underfilling or clotting of blood in the tube. Four additional animals were excluded because 2 each had PCV or HGB results that were slightly below the reference interval, resulting in a study population of 114 animals. There were 94 alpacas (52 females, 38 intact males, and 4 castrated males) and 20 llamas (13 females, 1 intact male, and 6 castrated males). The alpacas ranged in age from 1 to 15 years (mean, 5.5 years), and the llamas ranged in age from 3 to 16 years (mean, 10.4 years). There were 10 adult alpacas (3 females and 7 males) for which ages were unknown.

### 3.2 *M. haemolamae* and erythrocyte variables

**Figure 1:**
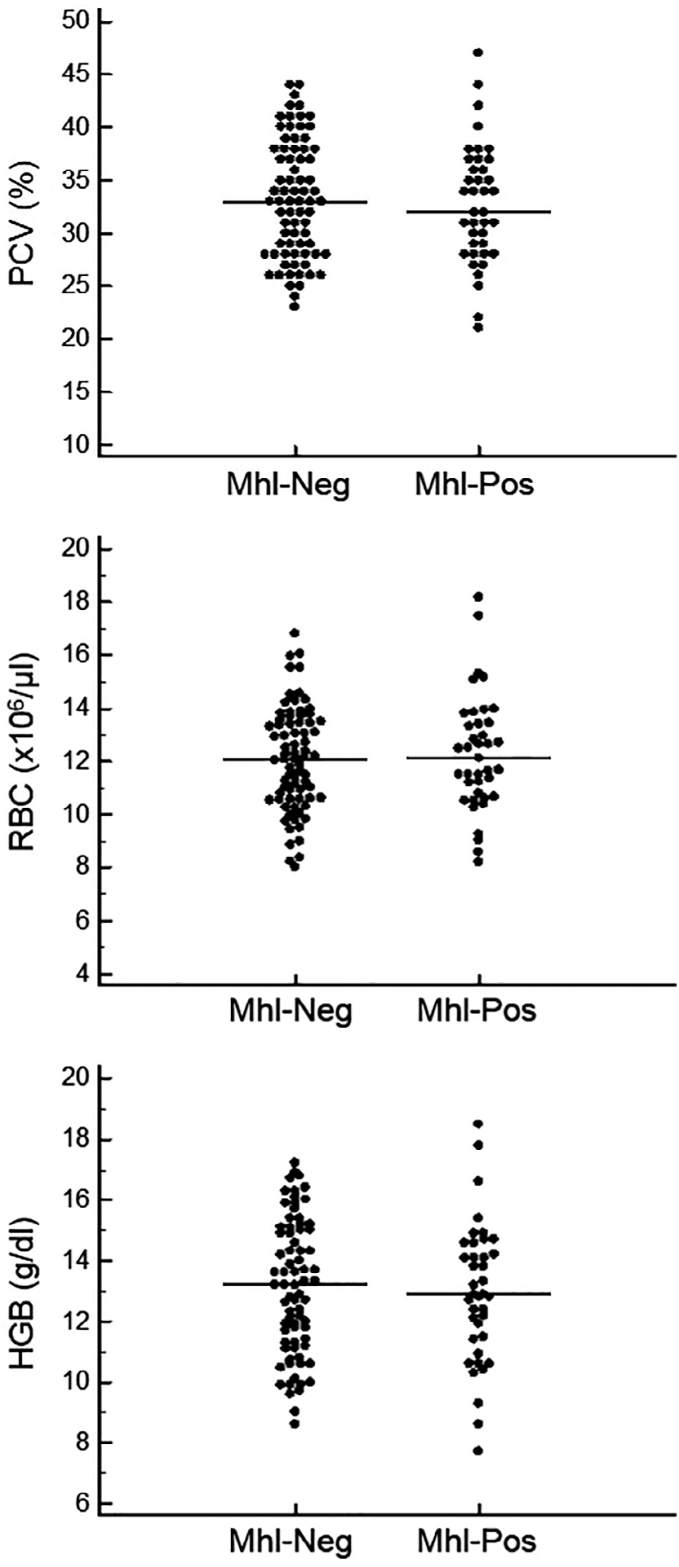
PCV, RBC count, and HGB concentration in camelids that were negative (Mhl‐Neg, n = 75) and positive (Mhl‐Pos, n = 39) for *Mycoplasma haemolamae* by real‐time PCR. The horizontal line indicates the median

A total of 39/114 animals (35 alpacas and 4 llamas) tested positive for Mhl on real‐time PCR, resulting in a 34.2% overall prevalence. The average per farm prevalence was 31.8%, with individual farm prevalence ranging from 0% (2 farms) to 100% (1 farm). No *Mycoplasma* organisms were observed on blood smears from any animal. Erythrocyte variables (PCV, RBC, HGB) for Mhl‐positive and Mhl‐negative animals were similar (Figure 1), and differences were not statistically significant. Median PCV for the Mhl‐negative group (n = 75) was 33.3% and for the Mhl‐positive group (n = 39) was 32.7% (*P* = .53). Median RBC count for the Mhl‐negative group was 12.09 ×10^6^/μL and for the Mhl‐positive group was 12.28 × 10^6^/μL (*P* = .98). Median HGB concentration for the Mhl‐negative group was 13.1 g/dL and for the Mhl‐positive group was 12.9 g/dL (*P* = .73). Erythrocyte variables also were compared between Mhl‐positive and Mhl‐negative animals within each FEC level (<500 epg, 500‐1000 epg, and > 1000 epg) to assess if a Mhl‐positive PCR result had an independent effect on erythrocyte variables. No statistically significant difference was found in the low FEC (<500 epg) group between Mhl‐negative and Mhl‐positive animals for PCV, RBC, or HGB (*P* = .74, *P* = .56, and *P* = .76, respectively). Too few Mhl‐positive animals were present in the moderate FEC group (n = 3) and high FEC group (n = 1) to allow statistical evaluation of the differences in erythrocyte variables based on Mhl PCR result within those subgroups.

### 3.3 Fecal egg count and erythrocyte variables

The FECs ranged from <50 to 3200 epg, with a mean and SD of 100 ± 405 epg. In addition to strongylid eggs, other nematode eggs (*Nematodirus* spp*., Aonchotheca [Capillaria]* spp.), coccidia (*Eimeria* spp.), and tapeworm eggs (*Moniezia* spp.) were noted in the McMaster chamber after fecal flotation; their presence was described qualitatively. Larval cultures were performed on 18/114 fecal samples, including samples from 5 different farms, and the L3 stage of *H. contortus* grew in all cultures. Additional culture findings included presence of *Trichostrongylus* spp. (4/18) and *Nematodirus* spp. (4/18).

**Figure 2:**
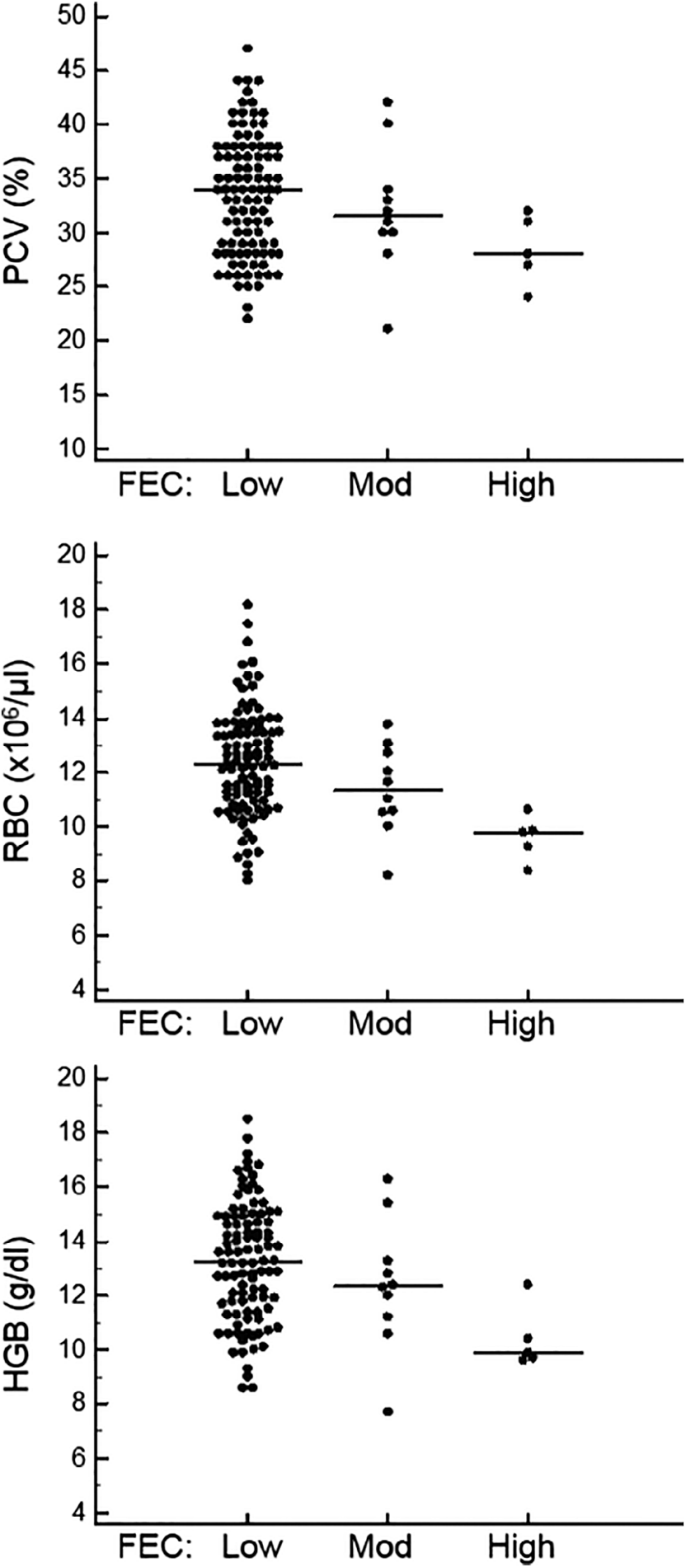
PCV, RBC count, and HGB concentration in camelids with low FEC (<500 epg, n = 99), moderate FEC (500‐1000 epg, n = 10), and high FEC (>1000 epg, n = 5). The horizontal line indicates the median

Animals were stratified into low (<500 epg), moderate (500‐1000 epg), and high (>1000 epg) FEC groups, and erythrocyte variables were compared among groups (Figure 2). The median PCV for each subgroup was 34% (low FEC), 31.5% (moderate FEC), and 28.0% (high FEC). The median RBC count was 12.33 × 10^6^/μL, 11.34 × 10^6^/μL, and 9.79 × 10^6^/μL for low, moderate, and high FEC, respectively. The median HGB concentration was 13.30 g/dL, 12.35 g/dL, and 9.90 g/dL for low, moderate, and high FEC, respectively. Statistically significant differences were noted between the low FEC and high FEC subgroups for PCV (*P* = .042), RBC (*P* = .0021), and HGB (*P* = .0039). The moderate FEC group was not statistically different from the low or high FEC groups for any erythrocyte variables.

**Figure 3:**
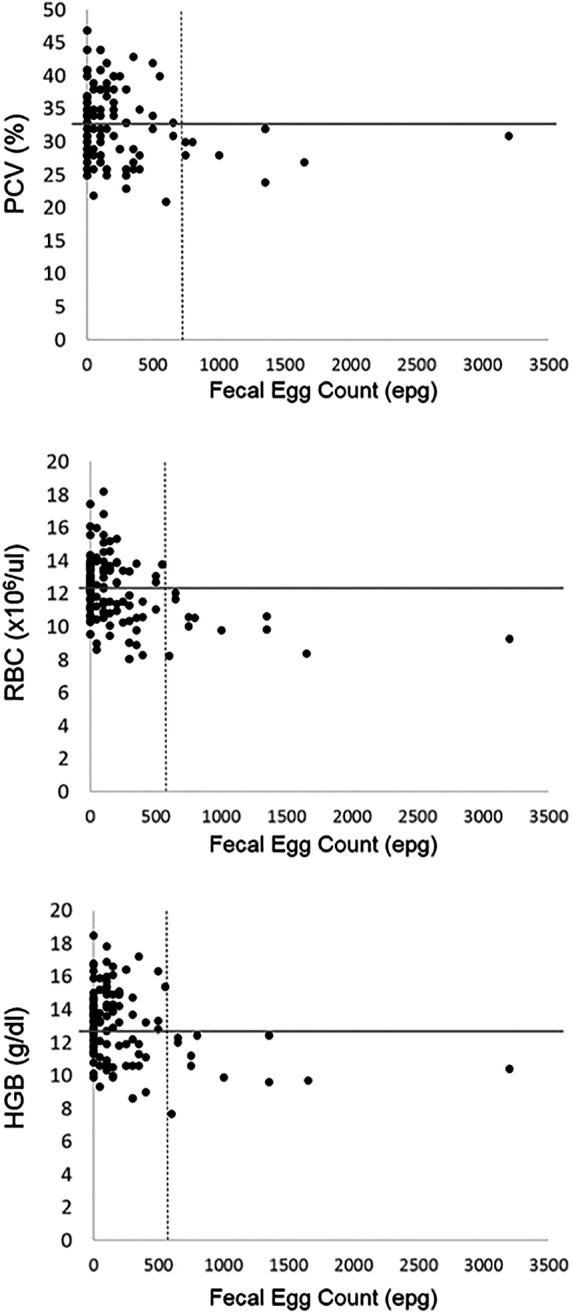
Scatterplot of erythrocyte variables (PCV, RBC, HGB) versus FEC. The horizontal gray line indicates the median of the reference interval. The vertical dashed line represents the FEC threshold above which all animals had an erythrocyte variable below the median of the reference interval. This threshold FEC was 750 for PCV and 600 for RBC and HGB

In concordance with the observation of decreasing erythrocyte variables with increasing FECs, scatterplots of FEC versus PCV, RBC, and HGB were visually inspected to determine if there was an FEC threshold above which all patient erythrocyte variables were below the median of the reference interval, suggesting that an FEC above that threshold may have a subclinical effect on erythrocyte variables. These thresholds were apparent at >750 epg for PCV and at >600 epg for RBC and HGB (Figure 3).

## 4 DISCUSSION

We found no statistically significant differences in mean erythrocyte variables (PCV, RBC, HGB) between *M. haemolamae*‐positive and *M. haemolamae*‐negative animals in a population of clinically healthy, nonanemic adult alpacas and llamas. Our results support the findings of previous research, suggesting that many cases of *M. haemolamae* infections are subclinical and that antimicrobial treatment is likely not warranted based on PCR status alone.^1^, ^7^ Interestingly, the prevalence of *M. haemolamae* infection in our study population (34.2%) is higher than that reported in previous prevalence studies for this organism in other geographic areas.^7^, ^14^, ^15^ This increased prevalence may reflect the subtropical latitude at which the current population resides (possibly supporting vector‐borne disease transmission), or different husbandry practices for camelids in various parts of the world. Ultimately, further study is needed to better understand the subclinical nature of *M. haemolamae* infections in camelids and the factors that allow for development of clinically relevant hemolytic anemia.

Our results indicate that clinically healthy, nonanemic animals with high FECs (>1000 epg) are more likely to have lower PCV, RBC, and HGB than animals with low FECs (<500 epg). Additionally, all study animals with FEC > 750 epg (8/114) had PCV below the median of the reference interval, and all with FEC > 600 epg (12/114) had RBC and HGB below the median of the reference intervals. Because these animals were clinically healthy, we would expect the erythrocyte variables of the group to span the entire range of the reference interval, as was the case for those with lower FEC. The finding that all animals with FEC >600 or > 750 epg were in the lower half of the reference intervals suggests that, although they were not anemic, these animals still may be experiencing some adverse hematologic effects as a result of their high trichostrongyle burden. The erythrocyte variable reference intervals used in this study, however, are specific to the instruments, methodologies, and reference population at our institution. Clinicians should interpret all patient results in the context of their laboratory's reference intervals.

Alpaca and llama owners and veterinarians currently have limited tools to assess the clinical relevance of gastrointestinal parasite burdens and the need for parasite treatment in healthy animals. They often are faced with the limited options of either only deworming clinically anemic animals (ie, those with high FAMACHA score and low BCS),^10^ or prophylactically deworming all animals on the farm (or a random subset). The problem with the former approach is that animals are already showing adverse clinical signs before treatment. In contrast, excessive deworming should be avoided because it can promote parasite resistance to anthelminthic medication. The identification of an FEC threshold above which erythrocyte variables are likely to be negatively impacted could inform treatment decisions, allowing for targeted deworming of those animals with clinically relevant nematode burdens before they become clinically anemic.

One limitation, however, is that FEC testing using the modified McMaster's test is relatively imprecise. Counts can vary substantially among observers, with different portions of the same fecal sample, and with variations in the concentration of the flotation solution, sample dilution, and volume of the counting chamber.^16^ Additionally, the correlation between trichostrongylid egg numbers in feces and adult worm burden of a particular species (ie, *Haemonchus* spp.) in the GI tract is uncertain. Our study only allowed for assessment of the association between FEC and erythrocyte variables at a single point in time. It therefore would be useful to perform a prospective cohort study in the future, to determine whether an FEC >600 epg or >750 epg is truly predictive for the development of anemia.

Additionally, the exclusion of alpacas and llamas <1 year old from our study precluded evaluation of the potential effects of *M. haemolamae* infection on erythrocyte variables in young animals. Given that cases of clinical anemia have been reported in young, potentially immunocompromised camelids,^6^, ^17^ the possibility that negative erythrocyte effects may be more likely to be observed in association with infection in animals <1 year old should be considered for future study. The inclusion of young camelids (< 1 year old) in any future studies also would be helpful to assess the effects of GI nematodes on erythrocyte variables in this age group. Similarly, inclusion of both clinically healthy and clinically ill animals (with high FAMACHA, low BCS, or both) would allow for comparison of *M. haemolamae* prevalence between these groups, and for more definitive assessment of the subclinical nature of *M. haemolamae* infection.

Additional limitations of our study included inability to exclude the effects of other factors on the measured erythrocyte variables. These variables reflect an animal's overall erythrocyte mass, which is affected by multiple factors including age, hydration status, nutrient balance, and renal and bone marrow function. Despite the presence of known causes of anemia (*Mycoplasma* spp., GI nematodes), it is difficult to attribute changes in erythrocyte variables (or lack thereof) solely to the presence or absence of these organisms. For example, decreased erythrocyte variables may reflect iron deficiency or the effects of inflammation on erythropoiesis, rather than blood loss from endoparasites.

In conclusion, our results confirm the findings of previous studies that demonstrated a lack of association between *M. haemolamae* infection and PCV in alpacas and llamas, and expand these findings to also include a lack of association with RBC or HGB. Overall, these findings suggest that treatment of clinically healthy *M. haemolamae* PCR‐positive animals is not warranted, although *M. haemolamae* infection cannot be ruled out as a contributing cause to the future development of clinically relevant anemia in these camelids. Finally, with additional research and method validation, the use of a FEC threshold of >600 epg or > 750 epg in clinically healthy alpacas and llamas may provide a component for guiding deworming strategies.

## CONFLICT OF INTEREST DECLARATION

Authors declare no conflict of interest.

## OFF‐LABEL ANTIMICROBIAL DECLARATION

Authors declare no off‐label use of antimicrobials.

## INSTITUTIONAL ANIMAL CARE AND USE COMMITTEE (IACUC) OR OTHER APPROVAL DECLARATION

This study was performed under University of Tennessee IACUC protocol 2298‐0914.

## HUMAN ETHICS APPROVAL DECLARATION

Authors declare human ethics approval was not needed for this study.
